# Impact of Cervical and Lumbar Spine Surgeries on National Football League (NFL) Player Performance and Return-to-Play Outcomes

**DOI:** 10.7759/cureus.85706

**Published:** 2025-06-10

**Authors:** Geoffrey R O'Malley, Patrick Pema, Paxton Sweeney, Syed Sarwar, Nicholas Cassimatis, John J Knightly, Ira Goldstein, Nitesh V Patel

**Affiliations:** 1 Department of Neurosurgery, Hackensack University Medical Center, Hackensack, USA; 2 Department of Neurosurgery, Hackensack Meridian Health, Hackensack, USA; 3 Department of Neurosurgery, Hackensack Meridian School of Medicine, Hackensack, USA; 4 Department of Neurosurgery, Rutgers University, Newark, USA; 5 Department of Neurosurgery, Jersey Shore University Medical Center, Neptune, USA

**Keywords:** cervical spine surgery, lumbar spine surgery, nfl, player performance, spine surgery

## Abstract

Introduction: American football players face a higher risk of spine injuries due to the sport's high-impact nature, especially in the lumbar and cervical spine regions. These injuries may require surgical interventions aimed at allowing athletes to return to the sport. However, the effects of these surgeries on players' performance and career longevity have yet to be comprehensively studied.

Objective: This study aims to evaluate the impact of spine surgeries on National Football League (NFL) players’ return-to-play rates and performance. We hypothesize that players undergoing lumbar surgeries would demonstrate greater performance improvement and return-to-play rates compared to those undergoing cervical surgeries, with differences influenced by player position and injury location.

Study design and methods: This is a retrospective cohort study (III) for which NFL injury reports from 2005 to 2022 were reviewed to identify players who had undergone spine surgery. Data collected included player position, return-to-play, and years played following the procedure. Performance metrics were gathered using Super Bowl wins and Pro Football Focus (PFF) player performance ratings. Statistical analysis was conducted using Python version 3.10.12 (Python Software Foundation, Wilmington, DE, USA) to evaluate differences in return-to-play rates, performance changes, and career duration post-surgery.

Results: The study identified 144 spine surgeries (77 lumbar, 67 cervical) among 136 players. Players who had lumbar surgery had a 61% return-to-play rate, with an average performance rating increase of 6.3%. In contrast, those who had cervical surgery had a 47% return-to-play rate and an average performance rating decrease of 5.8%. Lumbar surgeries were more common among linemen with higher BMIs, while cervical surgeries were more frequent in skill positions. Players with a history of lumbar surgeries were more likely to return to play than those without previous surgeries.

Conclusion: Spinal surgeries significantly impact the careers of NFL players. Lumbar surgeries show better outcomes in terms of return-to-play rates and performance improvements compared to cervical surgeries. The differences in surgical outcomes based on injury location and player position highlight the need for tailored rehabilitation protocols. This study provides valuable insights for medical practitioners, team management, and athletes, contributing to a broader understanding of the implications of spine surgeries in professional football.

## Introduction

The high-impact nature of sports such as American Football exposes athletes to an increased risk of spinal injuries, notably in the cervical and lumbar regions [1-6]. These injuries result from the biomechanical demands of the sport, where repetitive axial loading and high-impact tackles impart excessive strain on the spine [1,5]. These injuries vary in severity based on the age of the player, the body part affected, and prior injuries to both the level of concern and adjacent segments [7]. Disc herniations are especially prevalent and account for a significant proportion of injuries that ultimately require surgical intervention [4]. Despite the risk of re-injury, many players attempt to return to play, driven either by financial incentives or passion for the game. 

For non-athletes, numerous patient-reported outcomes such as the Oswestry disability index, the neck disability index (NDI), and the cervical ranges of motion (CROM) exist to quantify the level of disability patients face in their day-to-day lives as a result of their pain related to the spinal column or from radiculopathy [8,9]. At this point, no scale currently exists to assess National Football League (NFL) players’ on-field performance following cervical or lumbar injuries. Because the careers of NFL players are determined by on-field performance, those who have suffered from spine-related pain or disability have little information to determine how various treatment options will affect their competitiveness. 

Cervical and lumbar spine injuries are not uncommon in the NFL and often result in significant time missed for rehabilitation and even surgical intervention [4]. By using Pro Football Focus Data [10], this study presents a novel exploration into the comprehensive impact of spine injuries and subsequent surgeries on NFL players, extending beyond the conventional scope of procedural techniques and clinical outcomes. By bridging the gap between medical advancements and the practical implications for elite athletes, this study not only contributes to the broader understanding of spine injuries in the context of professional football but also offers valuable insights that can inform medical practitioners, team management, and the athletes themselves.

## Materials and methods

Study design

This is an observational study examining trends between NFL players who sustained injuries to the cervical or lumbar spines and underwent surgery during the 2005-2022 NFL seasons. Approval from the local institutional review board was not needed as this study did not meet the definitions of human subject research.

Data collection

Data was sourced from publicly available NFL injury reports from 2005 to 2022. The location of injury, etiology of the injury, and type of procedure were recorded. Data regarding players injury histories were sourced from NFL.com (National Football League Enterprises LLC.), ESPN.com (ESPN Enterprises, Inc.), FoxSports.com (Fox Media LLC), CBSSports.com (CBS Interactive Inc.), DraftSharks.com (Fantasy Football Draft Sharks, Inc.), SharpFootballAnalysis.com (Sharp Football Analysis, LLC), and RotoWire.com (GDC America Inc).

If the etiology of the injury or type of procedure was not confirmed, these variables were listed as unspecified. Player age, height, weight, and position were sourced from their official NFL roster of the season or the season prior to the surgery occurring, if the surgery occurred in the offseason. Return to play following surgery was determined if the player appeared in another regular-season NFL game following surgery. Information regarding years in the league before and after surgery, Pro Bowl and Super Bowl appearances was sourced from Pro-Football-Reference.com. Player performance data was sourced from Pro Football Focus (PFF) [10]. Pro Football Focus assigns grades to players based on their overall contribution to their team as determined by a team of analysts. The PFF grades of 60 are described as “average”, with above 80 being “good”, and 90 being “elite”.

Statistical analysis

Analytical evaluations and data visualizations were carried out using Python version 3.10.12 (Python Software Foundation, Wilmington, DE, USA) [11]. For mathematical computations, the Python NumPy library was used [12]. Data preprocessing and transformations were managed through the Python Pandas library [13]. To conduct primary statistical tests, such as the independent two-sample t-test and the two-way ANOVA, the SciPy [14] and Statsmodels [13] libraries of Python were used. A p-value less than 0.05 was considered statistically significant. 

Multivariate analysis

Multivariate statistical analyses were conducted using Python version 3.8.0. Linear regression models were used to analyze continuous outcomes, such as the percentage change in professional football performance (PFP) scores. The overall model fit was evaluated using the F-statistic and its corresponding p-value. Logistic regression was employed to model binary outcomes, specifically whether players returned to play post-surgery. The goodness of fit for the logistic models was assessed using the likelihood ratio test, with significance determined by the log-likelihood ratio p-value.

## Results

From a search of injury reports, 144 instances of spine surgery were identified across 136 players. The full player characteristics are described in Table 1. There were 77 instances of lumbar surgery across 71 players. The average age at the time of surgery was 26.8 years. The most frequent positions to undergo lumbar surgery were offensive and defensive linemen, whose positions comprised nearly half of all lumbar procedures. Full positional data by procedure type is described in Table 2. The most common reasons for lumbar surgery were disc herniations (58) and vertebral fractures (3). There were 15 surgeries in which there was no underlying cause clearly described for the procedure. In terms of procedures performed, 58 players underwent discectomies, five underwent fusions, and 14 procedures were unspecified.

**Table 1:** Injury information and outcomes of players who underwent surgery Values are presented as N (%). A p-value < 0.05 is considered significant. PFF: Pro Football Focus

Variable	Lumbar surgery		Cervical surgery			
	Mean/Total	SD/Percentage	Mean/Total	SD/Percentage	p-value	Stat
No. of surgeries (players)	77 (71)	53.5%	67 (64)	46.5%		
Age at time of surgery	26.8	2.93	27.6	3.56	0.14456	t=-1.4356
BMI	32.3	4.67	30.5	4.03	0.01303	t=2.441
Offense	38	49.4%	31	46.3%	0.83988	χ²=0.0408
Defense	36	46.8%	36	53.7%	0.50395	χ²=0.4466
Special teams	3	3.9%	0	0.0%	0.29496	χ²=1.0981
PFF rating season of injury	67.6	14.7	68.1	9.7	0.80749	t=-0.2345
Average pro bowls before surgery	0.6	1.1	0.8	2.1	0.48366	t=-0.8606
Average super bowls before surgery	0.1	0.4	0.4	0.7	0.00196	t=-2.4163
Prior spine surgery	12	15.6%	4	6.0%	0.11751	χ²=2.4502
Injury type						
Disc herniation	58	74.0%	46	59.7%	0.48108	χ²=0.4964
Fracture	3	3.9%	6	7.8%	0.365	χ²=0.8206
Spinal cord contusion	1	1.3%	1	1.3%	1.0	χ²=0.0
Other/unknown	15	20.8%	14	18.2%	0.99769	χ²=0
Procedure type						
Discectomy/foraminotomy	58	75.3%	8	10.4%	<0.00001	χ²=55.4518
Fusion	5	6.5%	30	39.0%	<0.00001	χ²=26.496
Other/unknown	14	18.2%	29	37.7%	0.00104	χ²=9.613
Returned to play	47	61.0%	31	40.3%	0.10813	χ²=2.5814

**Table 2:** Surgeries by position Values are presented as N (%). A p-value < 0.05 is considered significant.

Position	Lumbar surgery		Cervical surgery			
Position	Total	Percentage	Total	Percentage	p-value	χ²
Quarterback	2	2.63%	3	4.48%	0.5485	0.021
Running back	3	3.95%	11	16.42%	0.01242	4.94
Wide receiver	6	7.89%	7	10.45%	0.59612	0.0569
Tight end	9	11.84%	2	2.99%	0.0477	2.79
Offensive line	17	22.37%	7	10.45%	0.05744	2.82
Defensive line	19	25.00%	11	16.42%	0.20766	1.11
Linebacker	8	10.53%	12	17.91%	0.20408	1.06
Defensive back	8	10.53%	13	19.40%	0.13362	1.59
Other	4	5.26%	1	1.49%	0.22246	0.59

There were noted to be 12 instances of prior lumbar surgery (16%). Following lumbar surgery, players returned to play following 47 of 77 procedures (77%). Notably, of the 12 players who underwent surgery after a prior lumbar procedure, 10 returned to play (83%). The return-to-play rate of players with a history of prior lumbar surgery was significantly higher than those who had not undergone a prior operation (83% vs. 56%, p=0.02034). The overall years played after lumbar surgery were 2.24 years. There was no significant variation in years played following surgery between those with and without a history of lumbar surgery (2.9 vs. 2.1, p=0.284827).

Regarding cervical procedures, there were 67 instances of surgeries noted, encompassing 65 players. The average age at the time of surgery was 27.6 years. The most frequent positions to undergo cervical surgery were skill positions (quarterbacks, running Backs, wide receivers, and defensive backs), which comprised just over half of the positions of all players that underwent cervical procedures. The most common reasons for cervical surgery were disc herniations (46) and vertebral fractures (6). There were 14 surgeries in which there was no underlying cause clearly described for the procedure. In terms of procedures performed, 30 players underwent fusions, eight underwent discectomy/foraminotomy, and 29 procedures were unspecified. Only four of 67, or 6% of players, had undergone prior cervical surgeries.

Comparison of lumbar and cervical surgery player characteristics and outcomes

No significant variation was observed between the ages of players undergoing either cervical or lumbar procedures (26.8 vs. 27.6, p=0.14456). Additionally, similarities were found between the underlying pathologies requiring surgery. However, significant variations were seen in the positions of players that comprised each group. The lumbar surgery cohort was predominantly linemen, while the cervical surgery cohort was mostly players at skill positions. Correspondingly, the BMI of players at the season of surgery was noted to be significantly higher in the lumbar surgery cohort (32.3 vs. 30.5, p=0.01303). Overall player performance ratings were similar in the season prior to surgery between the lumbar and cervical cohort (67.6 vs. 68.1, p=0.80749). The cervical surgery cohort was observed to undergo significantly more fusion procedures than the lumbar surgery cohort (39% vs. 6%, p<0.00001). There was no significant difference between the average number of years played following surgery between the lumbar or cervical cohorts, though lumbar tended to be longer (2.2 vs. 1.3, p=0.0798764).

Performance outcomes

Performance ratings were available for 33 of 77 players who underwent lumbar surgeries. The full performance outcomes regarding lumbar procedures are described in Table 3. Overall, the average performance rating before lumbar surgery was 68, while in the season following lumbar surgery, the average performance rating was 71. The average change in performance rating players from the season before surgery to the season after surgery was 6.3%. Players undergoing lumbar surgery were separated based on above-average (n=12) and below-average (n=21) performance ratings before surgery. There was no significant difference in return-to-play between the two groups (92% vs. 71%, p=0.39108). Above-average players saw significantly less improvement in performance following surgery than below-average players (-10% vs. 18.22%, p<0.0001). There was no significant difference in years played following surgery between the two groups (3.8 vs. 2.9, p=0.39108). 

**Table 3:** Demographic information and outcomes of players undergoing lumbar surgery by season prior to injury PFF rating Values are presented as N (%). A p-value <0.05 is considered significant. PFF: Pro Football Focus

Variable	Total		Above average/Good/Elite (PFF 70+)		Average/Below average PFF (<70)		p-value	Stat
	Mean/Total	SD/Percentage	Mean/Total	SD/Percentage	Mean/Total	SD/Percentage		
No. of players	77	53.5%	12	36.4%	21	63.6%	0.0264	χ² = -51.701
Age at time of surgery	26.8	2.9	27.3	2.61	26.1	3.00	0.2293	t = 1.2023
BMI	32.3	4.7	30.6	1.96	31.4	3.59	0.4078	t = -0.8278
Prior lumbar surgery	12	15.6%	5	41.7%	5	23.81%	0.2846	χ² = 0.0
Offense	38	49.4%	7	58.3%	5	23.81%	0.0477	χ² = 11.3549
Defense	36	46.8%	5	41.7%	16	76.19%	0.0477	χ² = -53.5896
Special teams	3	3.9%	0	-	-	-	-	
Injury type								
Disc herniation	57	74.0%	11	91.67%	20	95.2%	0.6818	χ² = -26.7514
Fracture	3	3.9%	1	8.33%	0	0.0%	0.1802	χ² = 41.5859
Spinal cord contusion	1	1.3%	-	-	-		-	
Other/unknown	16	20.8%	0	0	1	4.8%	0.4413	χ² = -95.4703
Procedure type								
Discectomy/foraminotomy	58	75.3%	11	91.67%	20	95.24%	0.6818	χ² = -26.7471
Fusion	5	6.5%	1	8.33%	0	0	0.1802	χ² = 41.5859
Other/unknown	14	18.2%	-	0	1	4.8%	0.4413	χ² = -95.4703
Returned to play	47	61.0%	11	91.67%	15	71.43%	0.1707	χ² = -13.0241
Years played after surgery	2.24	2.84	3.77	2.84	2.85	3.17	0.3911	t = 0.8577
Average PFF before	67.62	14.69	83.78	7.14	58.38	8.44	0.0000	t = 9.1892
Average PFF after	70.80	13.71	75.27	12.64	68.18	14.32	0.1400	t = 1.4758

Of the 67 players who underwent cervical procedures, performance data were available for 26 players. The full performance outcomes regarding cervical procedures are described in Table 4. Overall, the average performance rating before cervical surgery was 68, while in the season following cervical surgery, the average performance rating was 66.7. The average change in performance rating for players from the season before surgery to the season after surgery was -5.8%. Similar to patients undergoing lumbar surgery, the cervical surgery cohort was separated into above-average (n=9) and below-average (n=17) baseline performance. There was no variation in return to play between the above-average and below-average (78% vs. 59%, p=0.33204). Unlike the lumbar surgery cohort, there was no variation in postoperative percentage change in performance in the cervical surgery cohort (-4.8% vs. -6.4%, p=0.6578). Additionally, players with above-average baseline performance saw a higher number of years played following cervical procedures than those with below-average performance (1.9 vs. 1.2, p=0.0491). Significantly, fewer players in the cervical surgery cohort saw performance rating improvement following surgery than in the lumbar surgery cohort (22% vs. 58%, p=0.01928). 

**Table 4:** Demographic information and outcomes of players undergoing cervical surgery by season prior to injury PFF rating Values are presented as N (%). A p-value <0.05 is considered significant. PFF: Pro Football Focus

Variable	Total		Above average/Good/Elite (PFF 70+)		Average/Below average PFF (<70)		p-value	Stat
	Mean/Total	SD/Percentage	Mean/Total	SD/Percentage	Mean/Total	SD/Percentage		
No. of players	67	46.5%	9	34.62%	17	65.38%	0.1707	χ² = -40.7922
Age at time of surgery	27.6	3.6	28.89	4.01	27.53	3.47	0.3762	t = 0.861
BMI	30.5	4.0	28.11	1.81	29.42	2.81	0.2196	t = -1.4392
Prior cervical surgery	4	6.0%	1	11.11%	1	5.88%	0.6312	t = 0.0
Offense	31	46.3%	5	55.56%	8	47.06%	0.6818	χ² = -13.7902
Defense	36	53.7%	4	44.44%	9	52.94%	0.6818	χ² = -25.5057
Special teams	0	0.0%		0.00		0		
Injury type								
Disc herniation	46	59.7%	8	88.9%	15	88.2%	0.9601	χ² = -19.153
Fracture	6	7.8%	0	0.0%	0	0.0%	-	
Spinal cord contusion	1	1.3%	0	0.0%	0	0.0%	-	
Other/unknown	14	18.2%	1	11.1%	2	11.8%	0.9601	χ² = -35.3471
Procedure type								
Discectomy/foraminotomy	8	10.4%	1	11.1%	5	29.4%	0.9601	χ² = -49.7924
Fusion	30	39.0%	6	66.7%	6	35.3%	0.1260	χ² = 0.0
Other/Unknown	29	37.7%	2	22.2%	6	35.3%	0.4902	χ² = -35.3471
Returned to play	31	40.3%	7	77.8%	10	58.8%	0.3320	χ² = -10.1365
Years played after surgery	1.3	1.9	2.9	2.82	1.2	1.4	0.0491	t = 1.7009
Average PFF before	68.1	9.7	77.99	8.40	62.86	5.2	0.0001	t = 4.9269
Average PFF after	66.7	12.3	76.19	11.09	60.61	8.9	0.0007	t = 3.6396

Multivariate analysis

After controlling for age, BMI, and the round the player was drafted, no significant difference was observed in the change in PFF values after spine surgery (F=2.774, p=0.0563). This relationship was further analyzed by type of surgery, revealing no significant differences in PFF changes after a lumbar discectomy (F=0.9370, p=0.444) or a cervical fusion (F=1.124, p=0.375).

After controlling for age, BMI, the round drafted, and contract value before surgery, a significant difference was observed in the likelihood of returning to play after spine surgery (p=0.020). When analyzed by type of surgery, no significant differences were found in return to play after a lumbar discectomy (p=0.1243) or a cervical fusion (p=0.1255). The regression analysis revealed significant differences in the change in PFP scores post-surgery, based on the players' pre-injury performance levels. Specifically, players with a pre-injury PFP score above 70 experienced a more significant decrease in performance post-surgery compared to players with scores of 70 or below (F=8.917, p=0.00470).

## Discussion

Injuries requiring spinal surgeries can have a substantial impact on the careers of professional football players, emphasizing the distinct outcomes between cervical and lumbar surgeries. Through a review of public records, this study identified 77 lumbar procedures and 67 cervical procedures among NFL players. Return-to-play in at least one NFL game was noted in 61% of the lumbar procedures and in 47% of cervical spine procedures. Of players who returned to play, there was no statistically significant difference in the postoperative percentage change in performance in either the cervical or lumbar group. 

The differences were notable: players with lumbar surgeries showed a robust return-to-play rate, which might reflect the more straightforward nature of lower spine recoveries. In contrast, injuries necessitating cervical surgeries seem to involve more complex issues, which could explain the varied recovery and performance outcomes. Procedure type could also play a role- for instance, players with cervical injuries were much more likely to undergo a fusion procedure. Additionally, the finding that players previously undergoing lumbar surgery had a higher likelihood of return-to-play suggests improvements in surgical decision-making or rehabilitation approaches for these injuries.

Performance outcomes post-surgery also presented noteworthy trends. Players undergoing cervical surgeries typically experience a decline in performance, highlighting the severe impact these injuries can have. In general, cervical injuries with myelopathy are far less likely to make a full recovery than patients with pure cervical or lumbar radiculopathy [15]. In comparison, some individuals recovering from back surgeries showed slight performance improvements, which may influence how teams and medical staff manage player recoveries and expectations. This suggests a potential need for specialized, position-specific rehabilitation protocols that acknowledge the unique demands placed on players’ bodies, differing significantly between those who experience cervical versus back issues.

Furthermore, this study illustrates that a player’s position and physical demands critically affect the success of surgical interventions and subsequent recovery. Particularly, the predominance of linemen undergoing back surgery and their higher BMIs suggest that player-specific factors like body type and on-field roles should be considered when developing post-surgery rehabilitation plans. Larger players such as linemen typically undergo chronic issues relating to wear and tear, largely in part to their roles with blocking and load bearing. The chronic course of their injuries may allow these players to play through injury until some point at which their condition becomes unbearable. The nature of their position and physical responsibilities is contrasted with those of lighter and faster players, who are more likely to experience collisions and high-impact events during competition. This fast-paced style of play allows for less of these individuals to play through injury and results in a more immediate need for treatment upon injury. These insights could lead to a reevaluation of existing medical and training practices, potentially fostering more personalized care strategies that enhance recovery outcomes for spine-related injuries in professional football.

Limitations

While using Pro Football Focus data to quantify player performance is the best gauge, this study was limited regarding the proportion of players for which data was available. Pro Football Focus, as an organization, was founded in 2006, and that year was also the first during which it was able to compile a complete set of data on players. Thus, the use of its data as a metric prohibits any quantification prior to 2006. Additionally, since the organization has grown in scope and employee number since its inception [16], recent years present a more thorough and accurate grading of players. 

Since the NFL is only required to report injury type, the specific procedure the player underwent, and its exact anatomic region was often unavailable. Many news sites from respective teams covered the players’ injury and recovery progress, but often lacked details regarding procedure type. This lack of specificity can be attributed to both teams and individual players’ concerns for privacy surrounding their health.

## Conclusions

This study provides a comprehensive analysis of the impact of spine injuries and subsequent surgeries on the performance and return to play rates of NFL players. The findings demonstrate significant differences in outcomes based on the kind of surgery, position of the player, and pre-injury performance levels. Notably, players who underwent lumbar surgery experienced greater return-to-play rates, and in some cases, performance improvements compared to those who underwent cervical surgeries. Those who underwent cervical surgery often experienced declines in performance. These findings highlight the importance of considering player-specific factors, such as position and physical demands, in the post-surgical rehabilitation protocols and rehabilitation strategies. This study provides valuable insights for medical practitioners, team management, and athletes considering spine surgery. 
